# The Efficacy of COVID-19 Vaccines in Chronic Kidney Disease and Kidney Transplantation Patients: A Narrative Review

**DOI:** 10.3390/vaccines9080885

**Published:** 2021-08-10

**Authors:** Yi-Chou Hou, Kuo-Cheng Lu, Ko-Lin Kuo

**Affiliations:** 1 Division of Nephrology, Department of Medicine, Cardinal-Tien Hospital, New Taipei City 231, Taiwan; athletics910@gmail.com; 2 School of Medicine, Fu Jen Catholic University, New Taipei City 242, Taiwan; 3 Division of Nephrology, Department of Medicine, Taipei Tzu Chi Hospital, Buddhist Tzu Chi Medical Foundation, New Taipei City 231, Taiwan; kuochenglu@gmail.com; 4 School of Medicine, Buddhist Tzu Chi University, Hualien 970, Taiwan

**Keywords:** chronic kidney disease, COVID-19, dialysis, kidney transplantation, SARS-CoV-2, vaccine

## Abstract

The SARS-CoV-2 (severe acute respiratory syndrome coronavirus 2) pandemic has posed a huge threat to global health because of its rapid spread and various mutant variants. Critical illness occurs in the elderly and vulnerable individuals, such as those with chronic kidney disease. The severity of SARS-CoV-2 infection is associated with the severity of chronic kidney disease (CKD)and even kidney transplantation (KT) because of the chronic use of immunosuppressive agents. To develop adaptive immunity against SARS-CoV-2, vaccination against the spike protein is important. Current phase III trials of vaccines against SARS-CoV-2 have not focused on a specific group of individuals, such as patients with CKD or those undergoing dialysis or kidney transplantation. Chronic use of immunosuppressive agents might disturb the immune response to the SARS-CoV-2 spike protein. On the basis of limited evidence, the immune compromised status of CKD patients might decrease neutralizing antibody development after a single dose of a specific vaccine. Boosting dosage more than the protocol might increase the titer of the neutralizing antibody in CKD patients. Further evidence is needed to understand the factors disturbing the immunogenicity of the SARS-CoV-2 vaccine, and CKD patients should receive the recommended dose of the SARS-CoV-2 vaccine due to their relatively immune compromised status.

## 1. Introduction

The coronavirus 19 disease (COVID-19) pandemic has had a huge impact on health and economics because of its rapid spread and multiple mutant variants since 2020 [1]. The spectrum of COVID-19 varies from asymptomatic carriers to multiorgan dysfunction, including acute respiratory distress syndrome and acute kidney injury. Severe acute respiratory syndrome coronavirus 2 (SARS-CoV-2), the etiological agent of COVID-19, enters cells via the interaction between its spike protein and angiotensin-converting enzyme 2 (ACE-2) and thereby induces systemic inflammation and organ dysfunction by activating the NF-κB-associated inflammasome and producing a downstream cytokine storm [2,3,4]. The high-risk groups for SARS-CoV-2 -mediated critical illness include those with obesity, diabetes mellitus, advanced age and chronic kidney disease [5,6]. Among the patients with chronic kidney disease (CKD), the percentage of patients with critical illness was higher than that in the other groups because of the multiple comorbidities and the impaired immune system in CKD patients with varying CKD stages and even in patients undergoing kidney transplantation (KT) [7]. Beyond isolation with adequate social distancing and personal protective equipment, vaccination is a cornerstone strategy for lowering the incidence of critical illness during the COVID-19 pandemic. Currently, the techniques for SARS-CoV-2 vaccines include targeting inactive SARS-CoV-2, vectors carrying the nucleic acid for the spike protein, attenuated virus, a protein fragment of SARS-CoV-2, messenger RNA of SARS-CoV-2 carried by lipidic microparticles or the DNA sequence carried by an adenovirus [8]. Currently, evidence for the protective efficacy of the SARS-CoV-2 vaccine for CKD patients is growing. The aim of this narrative review article is to summarize the threat of the COVID-19 pandemic to CKD patients and the efficacy of COVID-19 vaccines in CKD patients.

## 2. COVID-19 Pandemic and CKD: The Higher Incidence of Critical Illness and Acute Kidney Injury in Patients with CKD

SARS-CoV-2 enters the host cells via ACE2. ACE2 interacts with the transmembrane proteases serine 2 and CD-147, thereby facilitating the entry of SARS-CoV-2 into host cells. In CKD patients, excessive renin-angiotensin-aldosterone system (RAAS) activation is common, especially in CKD patients with diabetes mellitus, in whom the activity of RAAS is often not altered by RAAS inhibitors [9]. A meta-analysis found that CKD patients had a higher incidence of severe SARS-CoV-2 illness. Singh et al. reviewed the association of comorbidities and severe SARS-CoV-2 infection at the beginning of the COVID-19 pandemic. The prevalence of severe COVID-19 was up to 4% in CKD patients [10]. Cai et al. also demonstrated that the odds ratio for mortality in CKD patients was up to 5.81 (95% confidence interval 3.78–8.94) [11]. From the OpenSAFELY database from the UK, the hazard ratio for COVID-19-related death increased with the severity of CKD status [12]. The advanced age and multiple comorbidities often prevalent in CKD patients might place these patients at higher risk of critical illness. A meta-analysis of the literature also demonstrated that patients with CKD alone had an increased association with all-cause mortality compared with those without CKD [13]. Data from a global health research network called TriNetX (Cambridge, MA) showed that the incidence of hospitalization, ventilation and mortality (risk ratios 1.4–1.5, 1.5–2.0 and 1.4–1.8, respectively) for COVID-19 were all higher in CKD patients than in those without CKD, even after enrolling KT patients [14]. The risk of SARS-CoV-2 infection was high in end-stage renal disease (ESRD) patients [15]. Data from a retrospective study by a midsized national dialysis provider in the US showed that ESRD patients were at higher risk because of the characteristics of congregated settings, and the mortality of SARS-CoV-2 infection in ESRD was up to 24.9% to 31% because of the immunosuppressive status and comorbidities, including old age and other disorders, such as hypertension or diabetes mellitus [15,16].

Beyond mortality in CKD, acute kidney injury (AKI) is also common in SARS-CoV-2 infection. From the autopsy results in SARS-CoV-2-related death, the kidney was the second-most vulnerable organ in SARS-CoV-2 infection [17]. The renal tubules express ACE-2, which facilitates the entry of SARS-CoV-2 from the renal tubules or from the basolateral aspect via the vasculature with the assembly of CD-147 [18]. In this manner, the severity of acute kidney injury would be more prominent in critically ill patients. Beyond the compromised hemodynamic status, an analysis of multiple databases demonstrated that the comorbidities mediated by SARS-CoV-2 infection might contribute to the higher incidence of AKI in COVID-19 [19]. The contributors to the recovery of AKI in SARS-CoV-2 were limited to survivors of SARS-CoV-2 infection [20]. Beyond medications such as corticosteroids or interleukin-6 antagonists, the preventive approach involves enhancing vaccination.

## 3. COVID-19 Vaccines of Choice in Preventing CKD Patients from Critical Illness

**Figure 1:**
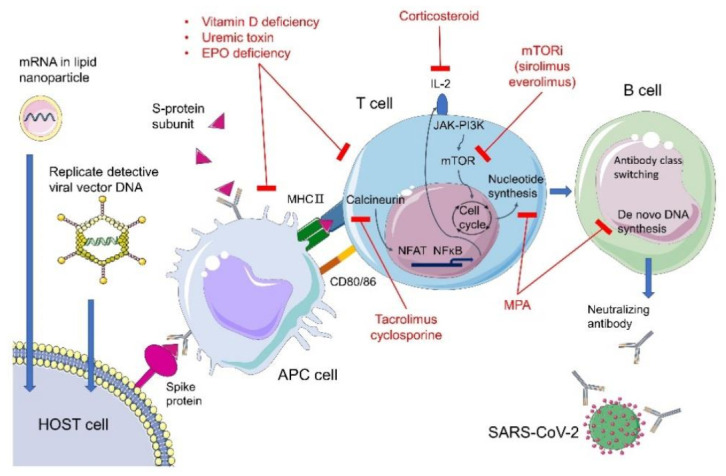
The mechanism of the impaired generation of neutralizing antibody after SARS-CoV-2 vaccine injection in CKD and KT patients. In CKD patients, the vitamin D deficiency, uremic toxin accumulation and erythropoietin deficiency could influence the antigen presenting ability of the antigen presenting cell (APC). In kidney transplantation (KT) patients, the corticosteroid, calcineurin inhibitor (tacrolimus or cyclosporin), mycopheloic acid (MPA) and mechanistic target of rapamycin (mTOR) inhibitor decrease the activity of T cells and the production of neutralizing antibody by B Cells in different aspects.IL2: interleukin 2; JAK-PI3K: Janus-activated kinase/phosphoinositide 3-kinase pathway; MHC: major histocompatibility complex.

The main aim of the COVID-19 vaccine is to establish immunity to the spike protein of SARS-CoV-2. For humans or mice, immunity to the S-protein could be induced by exposure to the full-length S-protein or the extracellular domain of the S-protein [21] (Figure 1). For SARS-CoV, a viral vector with the RNA expression receptor binding domain for the S protein served as the strategy for the development of the antibody. Moreover, for SARS-CoV, the S-protein of Tor2, GD03T13, and SZ3 within the S1 domain induced monoclonal antibodies with high affinity for the receptor binding protein for the S-protein [21]. In this manner, the neutralizing antibody could decrease the entry of SARS-CoV.

### 3.1. Replication-Defective Viral Vector Carrying Pathogen Gene(s)

In the SARS-CoV era, the most commonly used viral vectors included adenovirus, lentivirus, measle virus, poxvirus, rhabdovirus and alphavirus [22]. Adenovirus-based vectors have served as the most dominant vectors during COVID-19 vaccine development. ChAdOx-1 uses the full-length codon for the S-protein with a tissue plasminogen activator leader sequence. The prime-boosting strategy can elicit IgG against the spike protein [23]. AD26.COV. S, which uses adenovirus vector 26 with a full-length codon for the S-protein, is injected at a dose of 5 × 10^10^ viral particles and thereby induces an immune response [24]. Recombinant AD26 (rAd26) and AD5 (rAd5), which carry SARS-CoV-2 full-length glycoprotein S, are given at a dose of 10^11^ viral particles and thereby induce an immune response [25].

### 3.2. mRNA Vaccines

Both BNT162b2 and mRNA-1273 vaccines are lipid nanoparticle–encapsulated mRNA-based vaccines that encode perfusion-stabilized full-length spike proteins [26,27]. For BNT162b2, the full-length RNA with 2 mutation modified by proline can translate the locked-in perfusion transformation in order to augment the production of neutralizing antibody [27]. Based on the current evidence, the titer of neutralizing antibodies might be associated with protection against critical illness. Khoury et al. created a model for correlating the titer of neutralizing antibodies and the occurrence of severe disease related to SARS-CoV-2 [28]. The study demonstrated that the titer of Ab was negatively associated with severe disease. In the UK, vaccination with either ChAdOx1 or BNT162b2 decreased the occurrence of mild symptoms in individuals with COVID-19, and the protective effects of the vaccine increased in patients after receiving the second dose [29].

### 3.3. Purified Virus Components

Beyond the injection of mRNA coding the S-protein with nanoparticles or a viral vector, the subunit of S-protein has been used to induce the development of neutralizing antibodies against SARS-CoV-2 and to block viral entry via human ACE [30]. NVX-CoV2373, as the subunit of the S-protein, is derived from the established baculovirus *Spodoptera frugiperda* (Sf9) insect cell-expression system. The mutation in the S1/S2 cleavage site makes the protein resistant to protease cleavage and thereby able to bind to hACE with high affinity. The adjuvant matrix protein is formulated in the subunit. Such evidence demonstrated that the neutralizing antibody might be negatively associated with severe SARS-CoV-2 infection, and an adequate dose of vaccine was warranted for the prevention of symptomatic COVID-19 infection [28]. Among the current vaccines with a 50% protective neutralization level, the vaccines above can induce the development of essential antibodies. However, phase III clinical trials enrolling CKD patients are few [31]. Only 0.7% of all subjects enrolled in the BNT162b2 trial had chronic kidney disease [27]. The ongoing NVX-CoV2373 trial (NCT04611802) and mRNA-1273 trial have not revealed the percentage of CKD patients enrolled [26].

## 4. COVID-19 Vaccine Efficacy in CKD Patients: From CKD3-5 and CKD-5d to Post Kidney Transplantation 

In clinical trials, the efficacy of vaccination has been assessed in different stages of CKD. Glenn et al. summarized findings from ongoing clinical trials of COVID-19 vaccines involving CKD patients [31] (Table 1). Among current phase III studies, only 39.4% of enrollees had mild to moderate CKD. Regarding immunosuppressive agents, 93.5% of phase III studies excluded participants on immunosuppressive agents. Therefore, assessing the efficacy of the vaccine based on different stages might be difficult. Currently, phase III studies on BNT162b2 (Pfizer), mRNA-1273 (Moderna), AD26.COV.2 (Johnson & Johnson), Novavax and ChAdOx1 nCoV-19 (AstraZeneca) are being conducted. In patients with CKD stage 3–5, the primary etiologies of CKD might influence the efficacy of the vaccine for COVID-19 because immunosuppressive agents might in turn influence adaptive immunity and therefore influence the IgG titer after vaccination.

### 4.1. COVID-19 Vaccination for CKD 3-5 Patients Not on Immunosuppressive Agents

To establish community immunity, a policy on COVID-19 vaccination is important to prevent critical illness in SARS-CoV-2 infection. Impaired innate and adaptive immunity is common in CKD patients [38]. For innate immunity, impaired costimulatory molecules such as CD80/CD86 for antigen-presenting cells and dendritic cells are common in uremic patients [39]. In addition, the expression of the Toll-like receptor is also modified in the uremic milieu. Ando et al. demonstrated that the decreased expression of toll-like receptor 4 on monocytes decreased proinflammatory cytokine release in uremic patients [40]. Adaptive immunity, which involves memory and the immune response aroused from innate immunity, depends on the activation of innate immunity. Antibody production by B lymphocytes decreases with APC dysfunction and memory T cell apoptosis under uremic conditions [38,41]. The impairment of antigen-presenting ability leads the immune systems of CKD patients to be unable to recognize the pathogen and arouse downstream adaptive immunity. B cell lymphopenia is also an important phenomenon in CKD patients [42]. Signals related to B cell differentiation, such as B cell activating factor, were shown to be impaired in CKD patients; therefore, the generation of antibodies might be influenced [43]. From the study by Frasca et al., the efficacy of the influenza vaccine on B cells was negatively associated with age but not with underlying illnesses such as diabetes mellitus [44]. Therefore, the CKD status and advanced age might influence the efficacy of the vaccine.

In CKD patients, both insufficient erythropoietin (EPO) and vitamin D can dysregulate immunomodulation. EPO is produced mainly in perivascular interstitial fibroblasts [45], and its receptors are distributed in T cells and antigen-presenting cells [46]. The homodimers of the EPO receptor can inactivate the downstream interleukin 2 receptor and therefore modulate regulatory T cells. The heterodimers of EPO receptors also modulate NF-kB action and therefore modulate excessive inflammation [47]. EPO resistance and deficiency are common in CKD patients, and supplementation with EPO might be a conjunctive strategy in managing the cytokine storm in CKD patients with COVID-19 [48]. The EPO titer has been noticed to augment the efficacy of the vaccine in ESRD patients [49]. Therefore, the efficacy of vaccines might be reduced by insufficient EPO in CKD patients.

Vitamin D deficiency is common in CKD patients. Active vitamin D (1,25(OH)2D) is generated from 1-α hydroxylase in renal tubular cells. Vitamin D deficiency is worsened in CKD patients by decline in glomerular filtration rate, proteinuria, tubulointerstitial injury, and the therapeutic dose of the active form of vitamin D [50]. From a previous study by Lu et al., vitamin D deficiency was shown to be associated with insufficient antimicrobial activity because of insufficient generation of cathelicidin [51], which might reflect insufficient innate immunity. Vitamin D status was also shown to be associated with vaccine efficacy in CKD patients. Zitt et al. demonstrated that the seroconversion rate with hepatitis B vaccine was lower in CKD patients with serum vitamin D levels < 10 ng/mL [52].

In summary, in CKD patients not on immunosuppressive agents, the efficacy of vaccines might be hampered because of the alteration of innate immunity by the uremic milieu, older age, and vitamin D and EPO deficiency. Therapeutic supplementation with EPO and vitamin D in different stages of CKD might be essential for maintaining the efficacy of the COVID-19 vaccine, although evidence for this is insufficient [53,54,55].

### 4.2. COVID-19 Vaccination for CKD 3-5 Patients on Immunosuppressive Agents

In treating CKD with glomerulonephritis (GN), such as primary GN or GN mediated by autoimmune disease, glucocorticoids and other immunosuppressive agents are used [56]. For lupus nephritis, corticosteroids and other immunosuppressive agents, such as cyclophosphamide and mycophenolic acid, are used as maintenance therapies [57]. For nephrotic syndromes such as minimal change disease or focal segmental glomerulonephritis, a high dose of prednisolone, up to 1 mg/kg/day, might be used to lower the severity of proteinuria and the sequential risk for a decline in the glomerular filtration rate [56]. However, for subjects with chronic glucocorticoid use, the efficacy of the COVID-19 vaccine might be lessened. Deepak et al. demonstrated evidence that the chronic use of prednisolone (at a dose of 6.5 ± 5.8 mg) decreased the development of neutralizing antibodies after mRNA vaccination [58]. On the basis of experience with the hepatitis B virus vaccine, steroid use might decrease the titer of antibody developed [59]. Although the current recommendations for COVID-19 vaccination do not exclude subjects with chronic use of glucocorticoids, chronic use might decrease the generation of neutralizing antibodies; therefore, a booster vaccine or other vaccine strategies might need to be considered.

In patients with primary GN, relapsing disease was documented after SARS-CoV-2 vaccination. Negrea et al. reported recurrent gross hematuria in two patients with IgA nephropathy after they received the Moderna vaccine [60]. In this case report, the patients had chronic proteinuria in the range of 1 g/day. Macianti et al. also reported a relapse of minimal change disease after BNT162b2 vaccine [61]. The antibody neutralizing spike receptor-binding domains were mostly immunoglobin G and immunoglobin A [62]. Therefore, the neutralizing antibody might elicit an increase in the antibody targeting the glomerulus and therefore induce the relapse of disease.

### 4.3. COVID-19 Vaccination for CKD-5d Patients

The efficacy of COVID-19 vaccination for hemodialysis patients was determined by Grupper et al. The vaccine applied in this study was the Pfizer BNT162b2 vaccine (Pfizer Inc., New York, NY, USA)-BioNTech, Mainz, Germany) [63]. The study measured IgG after the completion of two injections. In the dialysis patients, the humoral response was lower than that in the control group. Ninety-six percent of the dialysis patients had a humoral response. However, the antibody titer was lower in the dialysis group. Older age and relative lymphopenia were associated with a lower humoral response. The efficacy of COVID-19 for peritoneal dialysis was studied by Rodríguez-Espinosa et al. The vaccine applied in this study was Moderna (ModernaTX, Inc., Cambridge, MA, USA) mRNA-1273. The IgG titer for the S-spike protein of SARS-CoV-2 increased after the first and second doses of the vaccine [36]. Regarding the efficacy of COVID-19 vaccines using an mRNA vector, Lesny et al. observed an IgG response after the first dose of ChAdOx1 nCoV-19/Oxford or BNT162b in a dialysis center. The target subjects included dialysis patients, staff within the dialysis unit and HD patients with a previous history of SARS-CoV-2 infection. The effect of the first dose of the vaccine on IgG response was low when compared with that induced by the previous infection. Such evidence indicates that a scheduled booster dose should be mandatory for ESRD patients [64]. Findings from the case study by Ma et al. showed that lymphocytes including T cells, Th cells, killer T cells, and NK cells all decreased in hemodialysis (HD) patients, and serum cytokine levels were lower in HD patients with SARS-CoV-2 infection. A lower cytokine storm has been suggested to be protective against SARS-CoV-2 in ESRD patients [65]. Labriona et al. demonstrated the variation of IgG against SARS-CoV-2 after 3 months of infection in a HD facility. The IgG level weaned gradually within 3 months, and such serologic changes might predict less efficacy of vaccination [66]. From this aspect, the impaired immune system in CKD patients might confront difficulty in establishing sufficient adaptive immunity against SARS-CoV-2. Several clinical trials exclude patients with CKD or those on maintenance dialysis because of the expected poor response to the vaccine [67].

### 4.4. COVID-19 in Renal Transplantation Patients: The Effect of Immunosuppressants on Vaccine Efficacy

In KT patients, sustained immunosuppressive agents after transplantation are essential for graft function. The dendritic cells of the host and donors are activated and interact with alloantigen-reactive naive T cells and central memory T cells in the secondary lymphoid organs. Dendritic cells costimulate the CD3 complex, and CD80 and CD86 on the surface of dendritic cells engage CD28 on T cells [68]. After transplantation, the immunosuppressive agents administered include interleukin 2 receptor antagonists. The mainstay maintenance immunosuppressive agents include corticosteroids, calcineurin inhibitors, mechanistic target of rapamycin (mTOR) inhibitors, antimetabolites and costimulatory blockers [69]. The suggested initial maintenance regimen includes tacrolimus and antimetabolites with or without corticosteroids [69]. When treating acute rejection, immunosuppressive agents can be administered based on the characteristics of cellular or antibody-mediated rejection [70]. For cellular rejection, high-dose corticosteroids or lymphocyte-depleting agents are commonly used. For antibody-mediated rejection, an anti-CD20 antibody or plasmapheresis are applied [71,72].

Because immunosuppressive agents are widely used in KT patients, recommendations for scheduled vaccination against vaccine-preventable diseases have been made, such as vaccination for influenza or viral hepatitis [73]. Based on current evidence for influenza vaccines, the vaccines might not induce an autoimmune reaction, and they increase the graft rejection rate [74,75]. Therefore, the safety of vaccination should outweigh the risk of graft dysfunction. In the US, an mRNA-based COVID-19 vaccine has been administered to solid organ transplantation recipients. Based on the study findings, transplantation rejection occurred in 1 of the 741 participants [76]. The American Society of Transplantation also suggested the administration of the vaccine ideally more than 2 weeks before transplantation or 1–6 months after transplantation [77]. Therefore, the vaccination policy should be safe. For mRNA vaccines, only 37.5% of kidney transplantation patients had a humoral response after injection [34]. However, the immunosuppressive agent regimen might influence the development of neutralizing antibodies. Based on a report by Kamar et al., the titer of the neutralizing antibody for S-protein would be sufficient after the 3rd dose of an mRNA vaccine in solid organ transplant recipients [35]. Therefore, the dose of SARS-CoV-2 vaccine needs to be augmented to maintain a sufficient humoral response.

#### 4.4.1. For the Recipients with Maintenance Use of Corticosteroids

A corticosteroid-based regimen for kidney transplant recipients is the first-line strategy to avoid rejection [78]. Corticosteroids act on glucocorticoid receptors on monocytes or macrophages and therefore have anti-inflammatory effects [79]. A high dose of glucocorticoids is used during the perioperative period and immediately after the operation [80]. In the maintenance stage, a tapered dosage of 0.05–0.1 mg/kg per day of methylprednisolone along with other immunosuppressive agents is used. On the basis of evidence for the efficacy of other vaccines, chronic exposure to prednisolone might not influence the efficacy. Lode et al. demonstrated that the efficacy of the pneumococcal vaccine was not influenced by the chronic use of prednisolone [81]. For patients with glomerulonephritis with chronic use of immunosuppressants, the COVID-19 vaccine is still suggested. The efficacy of the vaccine might be better during the maintenance stage after transplantation. A booster injection with more than the regular dose might be considered.

#### 4.4.2. For the Recipients with Maintenance Use of Calcineurin Inhibitors

Calcineurin inhibitors (CNI) are commonly used along with steroids for kidney transplant recipients. Calcineurin inhibitors bind to cyclophilin and inhibit calcineurin phosphatase in T cells, subsequently hampering T cell activation [68]. A report by Ilies et al. showed that subjects with maintenance use of CNIs had a less seropositive response after the 1st dose of an mRNA vaccine [82]. Therefore, the second dose should not be delayed in KT patients. The observational study of Rahamimov et al. found that the use of CNIs might influence the seroresponsiveness to an mRNA vaccine [83]. Among the patients with a serologic response, the percentage of users of tacrolimus or cyclosporine was similar between groups (*p* = 0.870). However, patients with higher serum concentrations of CNIs (s >7 ng/mL for tacrolimus and >150 ng/mL for cyclosporine) were more common among those without a serologic response. In this study group, the estimated glomerular filtration rate (eGFR) for those without a serologic response was lower than that for those with a serologic response after vaccination. Such evidence might demonstrate that recipients receiving a relatively higher dose of CNI might have lower predicted efficacy. The efficacy for the prevention of severe SARS-CoV-2 infection was not demonstrated in this study, but the report from Ali Hsuan et al. showed that the protection induced by the first dose of an mRNA vaccine was weakened in those receiving a CNI-based maintenance therapy regimen [84]. Several reports also advocate a possible protective role in reducing the cytokine storm after SARS-CoV-2 infection [85]. Based on the evidence above, CNI users should receive a SARS-CoV-2 vaccination, and a higher dosage of immunosuppressive agents and a lower GFR might predict a poor response to the vaccine. Further investigations on the compliance with vaccination guidelines and the dose of the vaccine in this population are warranted.

#### 4.4.3. For the Recipients with Maintenance Use of Antimetabolites

Mycophenolic acid (MPA) inhibits the synthesis of guanosine monophosphate nucleotides and therefore decreases the proliferation of B or T cells [61]. MPA can directly decrease the proliferation and maturation of T cell [86]. Beyond T cells, the maturation and differentiation of B cells are also disturbed by MPA. A study by Haneda et al. showed that MPA could influence early B cell proliferation [87,88]. In this manner, the production of antibodies would be decreased in MPA users. The effect of MPA on the neutralizing antibody titer induced by the SARS-CoV-2 vaccine was also observed. A study by Rahamimov et al. showed that a lower mycophenolic acid dose was associated with higher responsiveness to the SARS-CoV 2 vaccine [83]. Since MPA might directly influence B cell activity, MPA recipients should be advised accordingly before receiving the SARS-CoV-2 vaccine.

#### 4.4.4. For the Recipients with Maintenance Use of mTORis

The use of mTORis in solid organ transplantation patients is growing. The advantage the use of mTORis is the lower incidence of posttransplantation diabetes mellitus and malignancy and the lower nephrotoxicity than with the use of CNIs [89,90]. mTOR inhibitors downregulate the proliferation of T and B lymphocytes [83,87]. The study from Garcia Jr. et al. stated that the cytokine storm mediated by SARS-CoV-2 is highly dependent on the mTOR-phosphatidylinositol 3-kinase (PIK)- protein kinase B (Akt) pathway [91], although the exact mechanisms between viral proliferation and the signal triggering mTOR activation are still unknown. Therefore, mTORis might play a therapeutic role in SARS-CoV-2 infection by reducing the cytokine storm. Regarding the efficacy of vaccines, the influence of mTORis on neutralizing antibodies after SARS-CoV-2 vaccination might be hampered. However, the results from Rahamimov et al. did not demonstrate the nonresponsiveness of antibodies in mTORi users. More evidence for mTORi users among solid organ transplantation patients might be needed.

## 5. Conclusions

The SARS-CoV-2 pandemic has posed a huge threat for CKD patients because of the higher risk of mortality in CKD and higher risk of the development of acute kidney injury. Beyond therapeutic agents, vaccines for the development of neutralizing antibodies against SARS-CoV-2 represent an important intervention for the prevention of critical illness in CKD patients. However, the severity of CKD and the administration of immunosuppressive agents could influence the efficacy of SARS-CoV-2 vaccines. The use of antimetabolites might hamper the development of neutralizing antibodies. Based on the evidence above, CKD patients should receive regular vaccinations and even booster doses during the SARS-CoV-2 pandemic.

## Figures and Tables

**Table 1:** The SARS-CoV2 vaccine in CKD-5d and kidney transplantation (KT) in clinical trials and experiences.

	Type of Immune Response	Clinical Trial for CKD-5d	Experiences in CKD-5d	Clinical Trial for KT	Experiences in KT
Purified virus components					
NVX-CoV2373 (Novavax)	IgM/IgG	Excluded in phase 2 study (NCT04368988) Not excluded in phase 3 study (NCT04611802)	N/A	Chronic exposure with immunosuppressive agents excluded in phase III (NCT04611802)	N/A
Replication-defective viral vector carrying pathogen gene(s)					
ChAdOx1 nCoV-19 (Oxford-AstraZeneca)	IgM/IgG	For mild to moderate renal disease in phase2/3 study (NCT04400838)	70.6% antibody response after 1st dose when comparing with BNT162b2 (81.8%, *p* = 0.3) [32]	Excluded for immunosuppressant medication within the past 6 months in phase 2/3 study	N/A
Sputnik V (Gamaleya Research)	IgG, cell mediated immunity	NCT04805632	N/A	Excluded in phase 3 study (NCT04741061)	N/A
Ad26.COV2.S (Janssen)	IgA, cell mediated immunity	Excluded in phase II study (NCT04436276)	N/A	Excluded in phase II study (NCT04436276)	N/A
mRNA vaccines					
BNT162b2 (Pfizer-BioNTech)	IgM/IgG, IgA, cell-mediated immunity	Not excluded in phase III trial	Lower anti–spike antibody level for dialysis patients than health control (116.5 AU/mL vs 176.5 AU/mL, *p* < 0.01) [33]	Excluded in phase I and III if anticipating the need for immunosuppressive treatment within the next 6 months (NCT04368728, NCT04713553)	37.5% of kidney transplant recipients had humoral response [34] 3rd dosage of mRNA vaccine could improve immune-genecity [35]
mRNA-1273 (Moderna)	IgM/IgG, cell-mediated immunity	Not excluded in phase III trial (NCT04470427)	97% of seroconversion after 2 doses of the mRNA-1273 vaccine separated by a 28-day interval [36]	Excluded if using for corticosteroids ≥ 20 milligram (mg)/day (NCT04470427)	Patients treated with calcineurin inhibitors, mycophenolate mofetil, or steroids showed significantly lower anti–SARS-CoV-2 antibody titers [37]

## Data Availability

No new data were created or analyzed in this study.

## Abbreviations

ACE-2	angiotensin-converting enzyme 2
AKI	acute kidney injury
Akt	protein kinase B (PKB/AKT)
CD	Cluster differentiation
CKD	chronic kidney disease
CNI	Calcineurin inhibitors
COVID-19	coronavirus 19 disease
eGFR	estimated glomerular filtration rate
EPO	Erythropoietin
ESRD	end stage renal disease.
GN	glomerulonephritis
HD	hemodialysis
KT	kidney transplantation
MPA	Mycophenolic acid
mTOR	mechanistic target of rapamycin
NF-kB	nuclear factor kappa-light-chain-enhancer of activated B cells
PI3K	phosphatidylinositol 3-kinase
RAAS	renin-angiogenin-aldosterone system
SARS-CoV-2	severe acute respiratory syndrome coronavirus 2
